# Increased gene expression noise in human cancers is correlated with low p53 and immune activities as well as late stage cancer

**DOI:** 10.18632/oncotarget.12457

**Published:** 2016-10-04

**Authors:** Rongfei Han, Guanqun Huang, Yejun Wang, Yafei Xu, Yueming Hu, Wenqi Jiang, Tianfu Wang, Tian Xiao, Duo Zheng

**Affiliations:** ^1^ Shenzhen Key Laboratory of Translational Medicine of Tumor, Department of Cell Biology and Genetics, Shenzhen University Health Sciences Center, Shenzhen, Guangdong, 518060, P.R.China; ^2^ Guangdong Key Laboratory for Biomedical Measurements and Ultrasound Imaging, School of Biomedical Engineering, Shenzhen University Health Sciences Center, Shenzhen, Guangdong, 518060, P.R.China

**Keywords:** gene expression noise, p53, immune activity, cancer prognosis

## Abstract

Gene expression in metazoans is delicately organized. As genetic information transmits from DNA to RNA and protein, expression noise is inevitably generated. Recent studies begin to unveil the mechanisms of gene expression noise control, but the changes of gene expression precision in pathologic conditions like cancers are unknown. Here we analyzed the transcriptomic data of human breast, liver, lung and colon cancers, and found that the expression noise of more than 74.9% genes was increased in cancer tissues as compared to adjacent normal tissues. This suggested that gene expression precision controlling collapsed during cancer development. A set of 269 genes with noise increased more than 2-fold were identified across different cancer types. These genes were involved in cell adhesion, catalytic and metabolic functions, implying the vulnerability of deregulation of these processes in cancers. We also observed a tendency of increased expression noise in patients with low p53 and immune activity in breast, liver and lung caners but not in colon cancers, which indicated the contributions of p53 signaling and host immune surveillance to gene expression noise in cancers. Moreover, more than 53.7% genes had increased noise in patients with late stage than early stage cancers, suggesting that gene expression precision was associated with cancer outcome. Together, these results provided genomic scale explorations of gene expression noise control in human cancers.

## INTRODUCTION

All the processes of life depend on spatially and temporally controlled gene expression. In individual cells, transcription is a process that often occurs in a bursty, intermittent manner [1, 2]. The frequency and size of these bursts affect the magnitude of temporal fluctuations in messenger RNA and protein content within a cell, creating variation or noise in gene expression [3]. Even in genetically identical cells, gene expression noise exists due to intrinsic and extrinsic factors. Intrinsic noise is generated as the inherent consequence of stochastic fluctuations in biochemical reactions whereas extrinsic noise is from extrinsic sources such as cell-to-cell fluctuations of transcription factors or from environmental diversity [4–7]. For example, nuclear architecture, chromatin modification, transcriptional dynamics at a promoter site, translation rates, mRNA degradation and protein degradation are sources of intrinsic noise [8]. Extrinsic noise may generate from availability of gene expression machineries, micro-fluctuations in cellular environment, cell division or asymmetric partitioning [8].

Expression noise can give rise to sub-populations of cells that rapidly respond to changing environmental stimuli. Such division of labor may be advantageous to modulate their function on a rapid time-scale. On the other hand, noise in gene expression may fundamentally limit the accuracy of cellular processes, and thus should be minimized and compensated [9]. Several mechanisms of buffering noise in mammalian gene expression have been proposed, mostly involving gene-specific solutions such as feedback or feed-forward motifs in their transcriptional regulation [10, 11]. Recently, Halpern et al. [12] combined deep sequencing of nuclear and cytoplasmic RNA fractions with single-molecule transcript imaging in mammalian cells and demonstrated that nuclear retention of mRNA could efficiently buffer cytoplasmic transcript levels from noise that emanated from transcriptional bursts. By using single-cell reporter assays, Schmiedel et al. [13] showed that microRNAs decreased protein expression noise for lowly expressed genes but increased noise for highly expressed genes. The authors estimated that hundreds of (lowly expressed) genes in mouse embryonic stem cells had reduced noise due to substantial miRNA regulation. Their findings suggested that microRNAs conferred precision to protein expression. However, studies on the control of gene expression noise at population level were relatively lack.

It is well recognized that human cancer is heterogenous [14, 15]. Numerous genetic lesions are involved in cancer development, together with abnormalities in DNA methylation, histone modification, promoter accessibility and other genome-wide rewirings, which result in expression deregulation of many genes [16–18]. Even in the same tumor, intra-tumoral genetic heterogeneity has been revealed by sequencing of the genomes of cancer cells from different sectors [19, 20]. In addition to this, tumor infiltrated endothelial, stromal and immune cells add more complex to gene expression variability in human cancers [21]. On the other hand, gene expression noise gives cell the environmental adaptation and evolution advantages under adverse conditions [22]. However, the regulation/deregulation of gene expression noise in human cancers and its underlying mechanism (s) were not determined. The Cancer Genome Atlas project sequenced mRNA transcripts of ample human cancer patients, which offered us the opportunity to probe the gene expression noise change between matched tumor-normal tissues at population level and its significance in cancer development.

## RESULTS

### Gene expression noise was increased in human cancers

**Figure 1:**
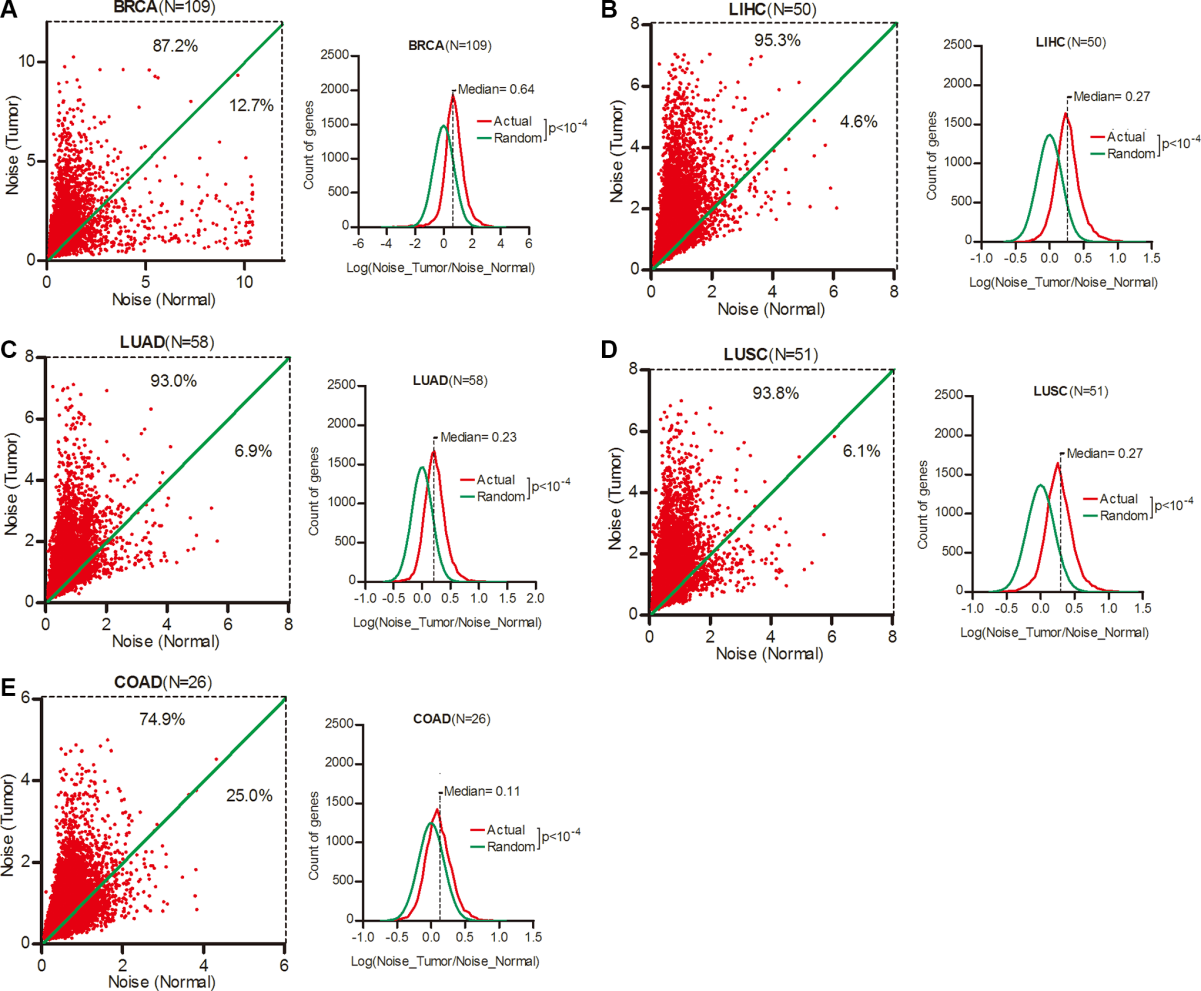
Gene expression noise was increased in human cancers (**A–E**) The expression noise was calculated as STDEV/AVERAGE in tumor or normal tissues for each of a total of 16,424 genes. The frequency of Log (Noise_Tumor/Noise_Normal) was plotted in contrast to random distribution. Wilcoxon's signed rank test was used to test whether the median of Log (Noise_Tumor/Noise_Normal) equals to 0. BRCA, breast invasive carcinoma; LIHC, liver hepatocellular carcinoma; LUAD, lung adenocarcinoma; LUSC, lung squamous cell carcinoma; COAD, colon adenocarcinoma.

We took breast invasive carcinoma (BRCA), liver hepatocellular carcinoma (LIHC), lung adenocarcinoma (LUAD), lung squamous cell carcinoma (LUSC) and colon adenocarcinoma (COAD) as our research objects due to their high incidence worldwide. For each of a total of 16,424 genes, we calculated its expression noise (defined as Standard Deviation divided by Average [13]) in tumor and paired normal tissues of different cancer types (Supplementary Table S1), and found that 87.2%, 95.3%, 93.0% and 93.8% of the 16,424 genes had increased expression noise in tumor tissues in BRCA, LIHC, LUAD and LUSC respectively (Figure 1A–1D, left). To a lesser extent, the ratio of genes with increased expression noise in tumor tissues was 74.9% in COAD (Figure 1E, left). We calculated the Log (Noise_Tumor/Noise_Normal) value of each gene in different cancer types, and plotted the frequency at the values in contrast to random distribution. Wilcoxon's signed rank test showed that the median of Log (Noise_Tumor/Noise_Normal) was significantly larger than zero in BRCA, LIHC, LUAD, LUSC and COAD (Figure 1A–1E, right), demonstrating that the gene expression noise was significantly increased in tumors when compared to normal tissues.

**Figure 2:**
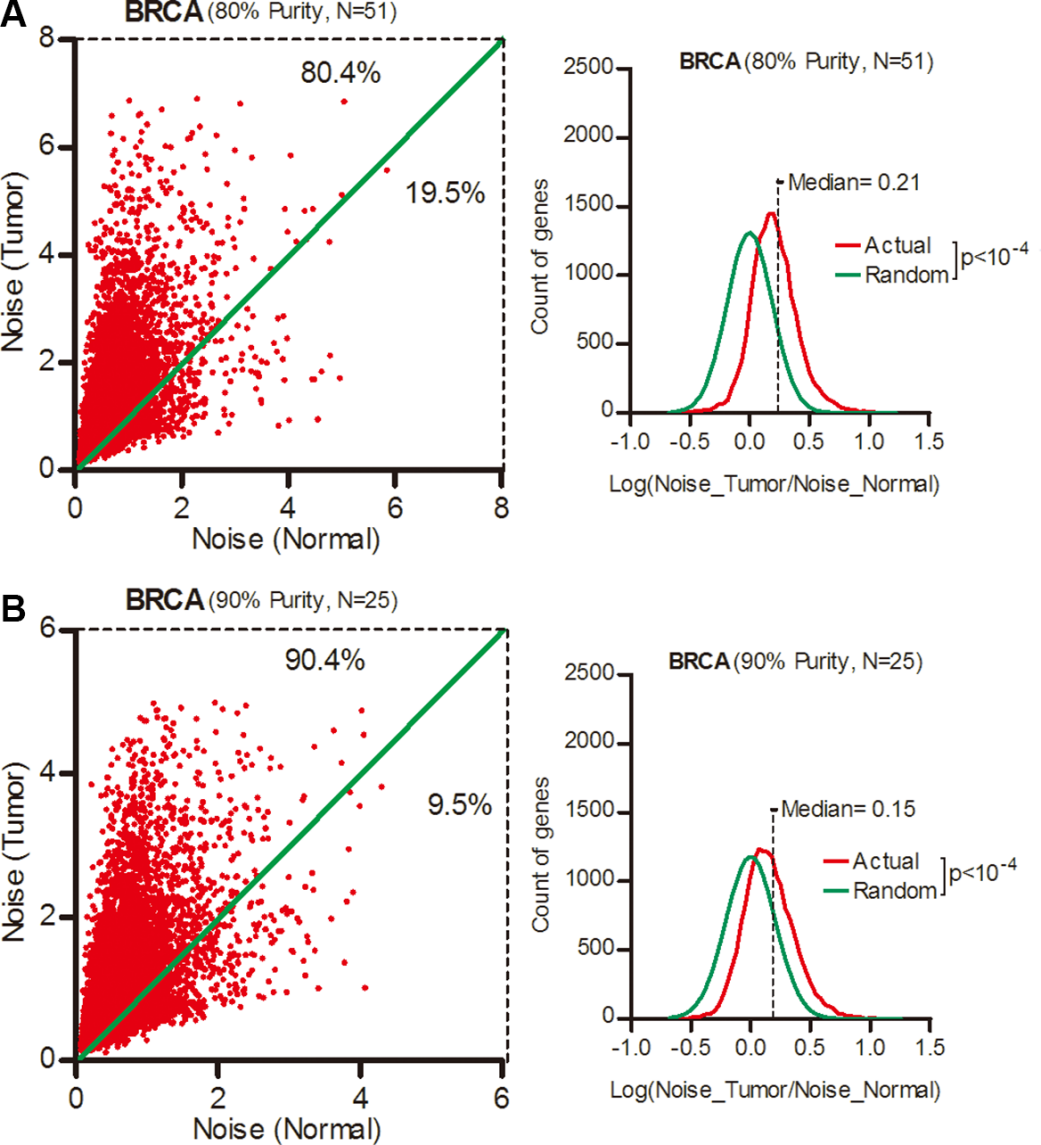
Gene expression noise increased in *ultra-pure* breast cancer samples Expression noise of each gene in tumor and normal tissues from breast cancer patients with tumor cell purity larger than 80% (**A**) or 90% (**B**) was shown as scatter plots. The frequency of Log (Noise_Tumor/Noise_Normal) was plotted in contrast to random distribution. Wilcoxon's signed rank test was used to test whether the median of Log (Noise_Tumor/Noise_Normal) equals to 0.

One possibility was that the increased noise arise from a mixture of tumor and infiltrated non-tumor cells. Therefore, we selected breast cancer patient for tumor cell purity larger than 80% or 90% according to their clinical data. Each subgroup contained 51 or 25 cases (Supplementary Table S2). In these two *ultra-pure* patient subgroups, there were still more than 80% genes with increased expression noise in cancer tissues (Figure 2A and 2B, Wilcoxon's signed rank test *p* < 0.0001), although we noticed a tendency of greater expression noise in patients with less tumor purity. Thus, the increased expression noise in tumor tissues was not caused by mixture of non-tumor cells.

### A common gene set with increased expression noise existed across different cancer types

**Figure 3:**
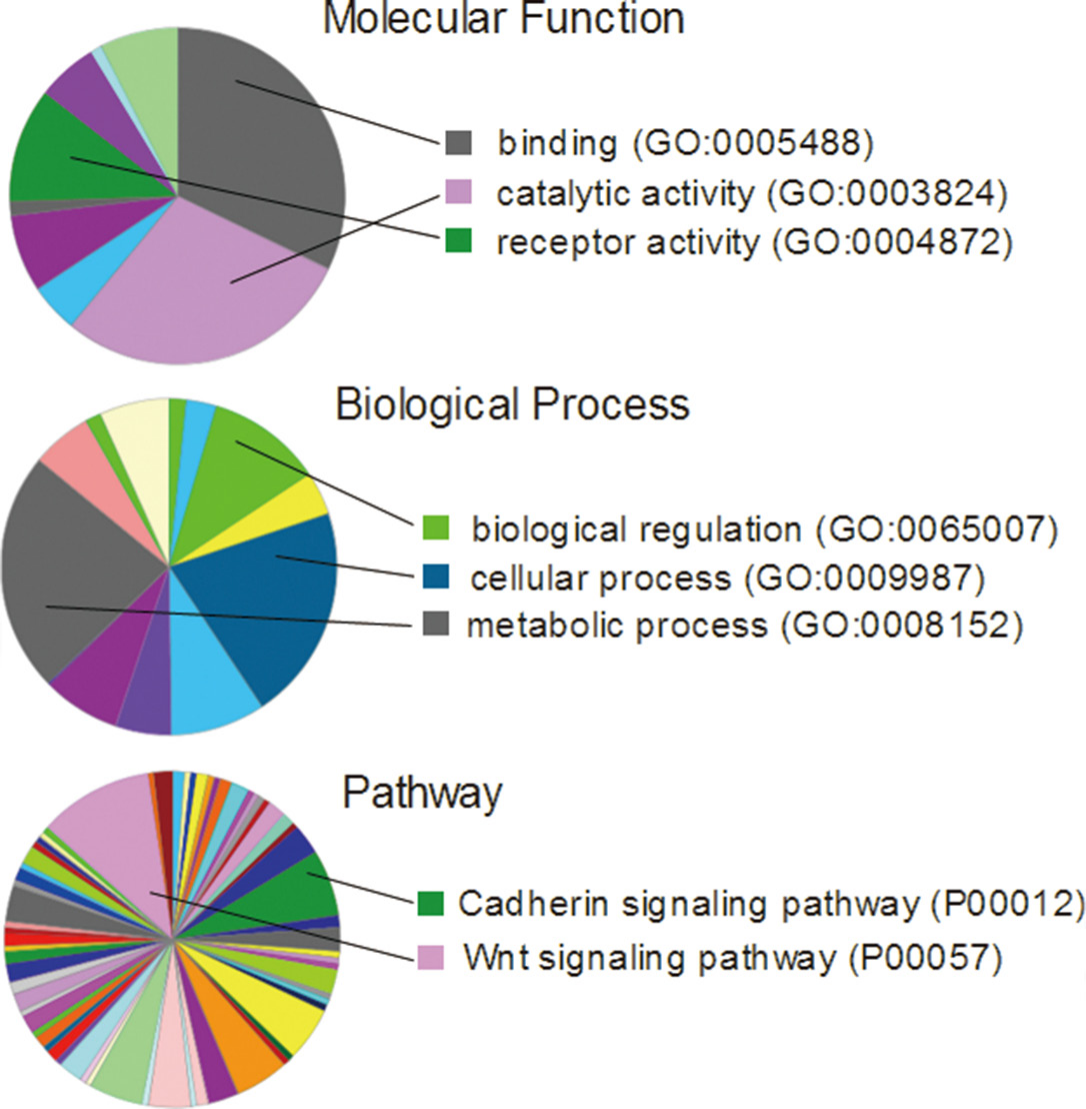
Gene ontology analysis of the 269 genes that had 2-fold increase of expression noise in BRCA, LIHC, LUAD, LUSC and COAD

We next took a closer look at the gene expression noise in cancers. There were 9,160 genes with increased expression noise shared by BRAC, LIHC, LUAD, LUSC and COAD. When we set the threshold of noise fold change (Noise_Tumor/Noise_Normal) > 1.5 or > 2, we retrieved 1,988 and 269 genes respectively (Supplementary Table S3). We performed Gene Ontology analysis of this set of 269 genes on PANTHER Classification System (http://pantherdb.org/), and found that they were clustered in cell adhesion, catalytic, metabolic and other functions, reflecting that these processes were most easily loss of control among cancer patients (Figure 3). We also tried to find the common genes that had decreased expression noise in tumor tissues of different cancer types. However, there were only 24 genes with decreased expression noise in BRCA, LIHC, LUAD, LUSC and COAD simultaneously, and 9 genes with expression noise decreased above 10% (Supplementary Table S3). The result further suggested that increased rather than decreased gene expression noise were much more frequent events in human cancers.

### Gene expression noise was inversely correlated with p53 status

**Figure 4:**
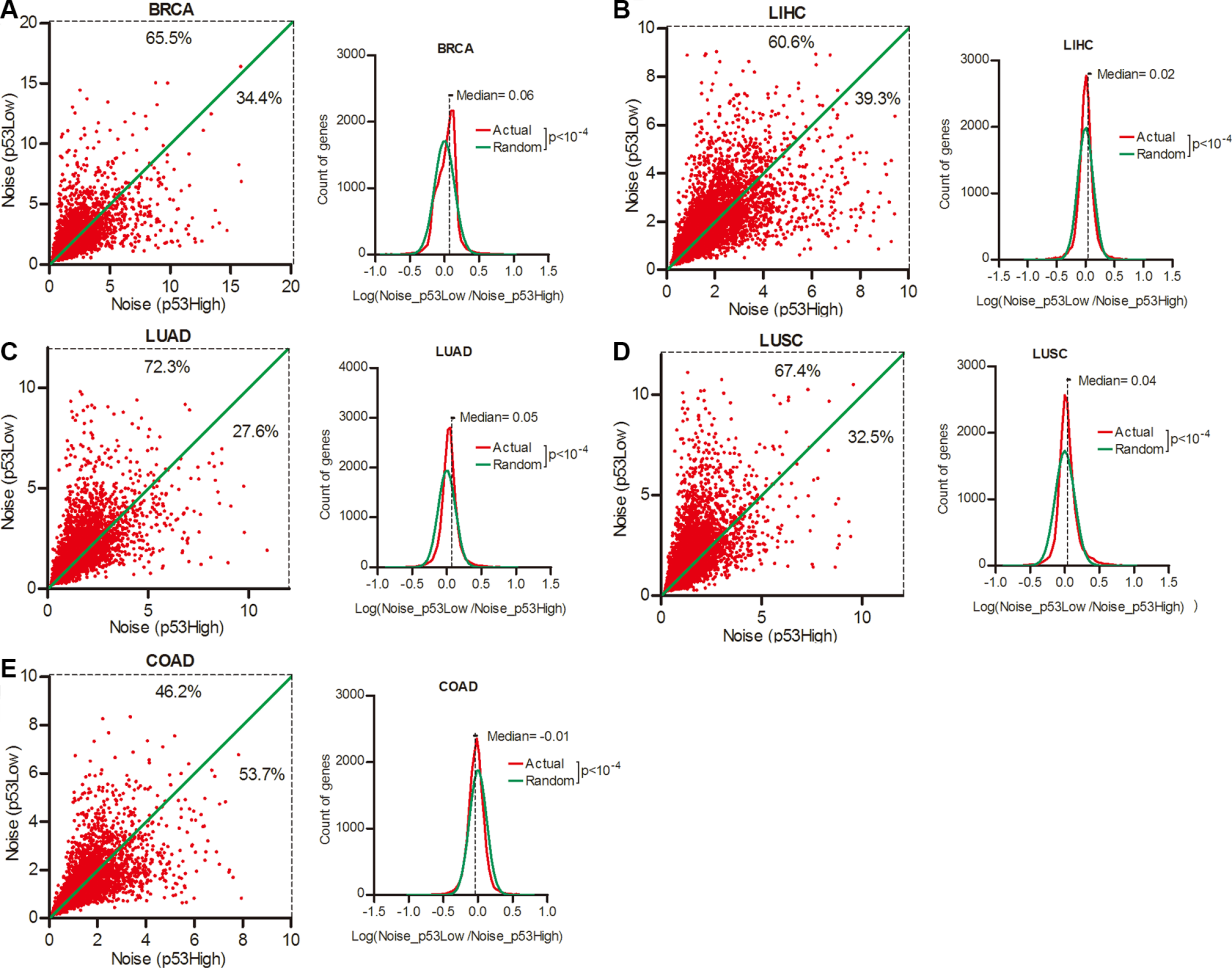
Gene expression noise was inversely correlated to p53 status in cancers Patient cohorts of BRCA (**A**), LIHC (**B**), LUAD (**C**), LUSC (**D**) and COAD (**E**) were sorted into the top and bottom quartiles of p53 activity in tumors. The gene expression noise in tumors was calculated in each quartiles. The frequency of Log (Noise_p53Low/Noise_p53High) was plotted in contrast to random distribution. Wilcoxon's signed rank test was used to test whether the median of Log (Noise_Tumor/Noise_Normal) equals to 0.

The p53 signal pathway is pivotal in maintaining genome stability, and is one of the most frequently mutated targets in cancer development [23]. We next investigated the contribution of p53 status to gene expression noise. Patient cohorts of different cancer types were sorted into the top and bottom quartiles of tumor p53 activity (Supplementary Figure S1 and Supplementary Table S4). We compared the expression noise of each of the 16,424 genes in lower and higher p53 activity groups, and observed that 65.5%, 60.6%, 72.3%, and 67.4% genes had increased expression noise in patient groups of lower p53 activity groups in BRCA, LIHC, LUAD and LUSC respectively (Figure 4A–4D, Wilcoxon's signed rank test *p* < 0.0001). These results suggested an inverse correlation between gene expression noise and p53 activity. But in COAD, less than a half (46.2%) of genes had increased expression noise in lower p53 activity group when compared to higher p53 activity group. (Figure 4E), suggested a different role of p53 pathway in gene expression noise control in COAD.

### Gene expression noise was inversely correlated with local immune activity

**Figure 5:**
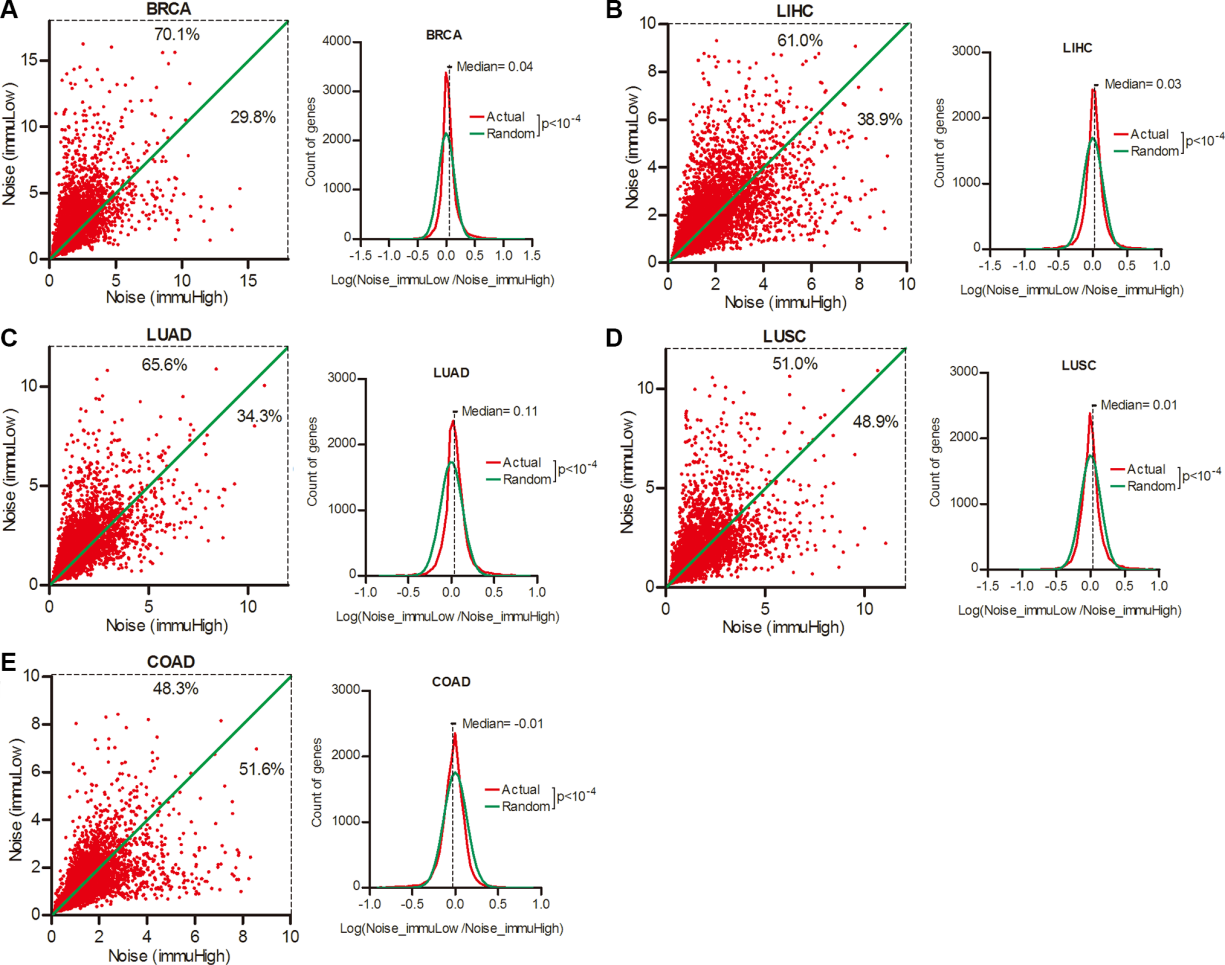
Gene expression noise was inversely correlated to local immune activity in cancers Patient cohorts of BRCA (**A**), LIHC (**B**), LUAD (**C**), LUSC (**D**) and COAD (**E**) were sorted into the top and bottom quartiles of immune activity in tumors. The gene expression noise in tumors was calculated in each quartiles. The frequency of Log (Noise_immuLow/Noise_immuHigh) was plotted in contrast to random distribution. Wilcoxon's signed rank test was used to test whether the median of Log (Noise_Tumor/Noise_Normal) equals to 0.

Host immune system has the potential to eliminate neoplastic cells. But its contribution in modulating gene expression noise in cancer is unknown. To address this issue, we sorted the patients of each cancer type into the top and bottom quartiles according to local immune activity in tumors (Supplementary Figure S2 and Supplementary Table S5), and compared the expression noise in lower and higher immune activity patient groups. As shown in Figure 5A–5D, 70.1% genes had increased noise in patient group of lower local immune activity in BRCA, followed by LIHC (61.0%), LUAD (65.6%) and LUSC (51.0%) (Wilcoxon's signed rank test *p* < 0.0001), with an exception in COAD (48.3%) (Figure 5E). Thus, gene expression noise was inversely correlated to local immune activity in BRCA, LIHC, LUAD and LUSC, but not in COAD.

### Smaller gene expression noise was associated with better patient prognosis

**Figure 6:**
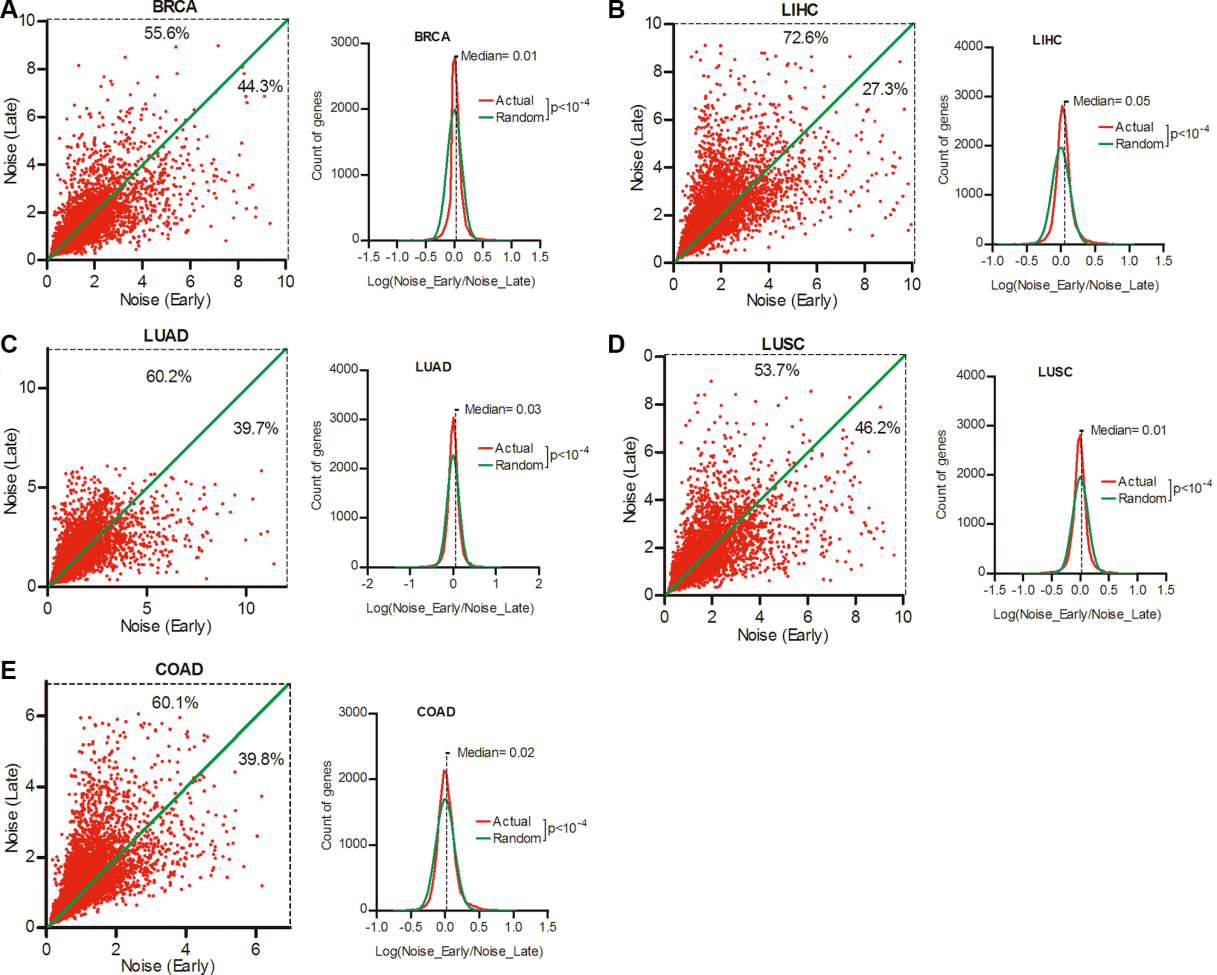
Smaller gene expression noise was associated with better patient prognosis Patient cohorts of BRCA (**A**), LIHC (**B**), LUAD (**C**), LUSC (**D**) and COAD (**E**) were divided into two groups, on include stage I at diagnosis(Early), the other include III or IV (Late). The expression noise was calculated in each groups. The frequency of Log (Noise_Late/Noise_Early) was plotted in contrast to random distribution. Wilcoxon's signed rank test was used to test whether the median of Log (Noise_Tumor/Noise_Normal) equals to 0.

p53 status and host immune activity are predictors of cancer prognosis [24]. Hence, gene expression noise may associate with disease outcome. To verify this hypothesis, we divided patients of each cancer type into two groups, one include stage I (early), the other include III or IV (late) at diagnosis (Supplementary Table S6), and compared gene expression noise between these two groups. Our data showed that 55.6%, 72.6%, 60.2%, 53.7% and 60.1% genes had increased expression noise in late stage as compared to early stage patients of BRCA, LIHC, LUAD, LUSC and COAD respectively (Figure 6A–6E, Wilcoxon's signed rank test *p* < 0.0001), suggesting that patient groups with better prognosis (early stage at diagnosis) tend to have smaller expression noise.

### Gene expression noise was positively correlated with p53 status in normal tissues

**Figure 7:**
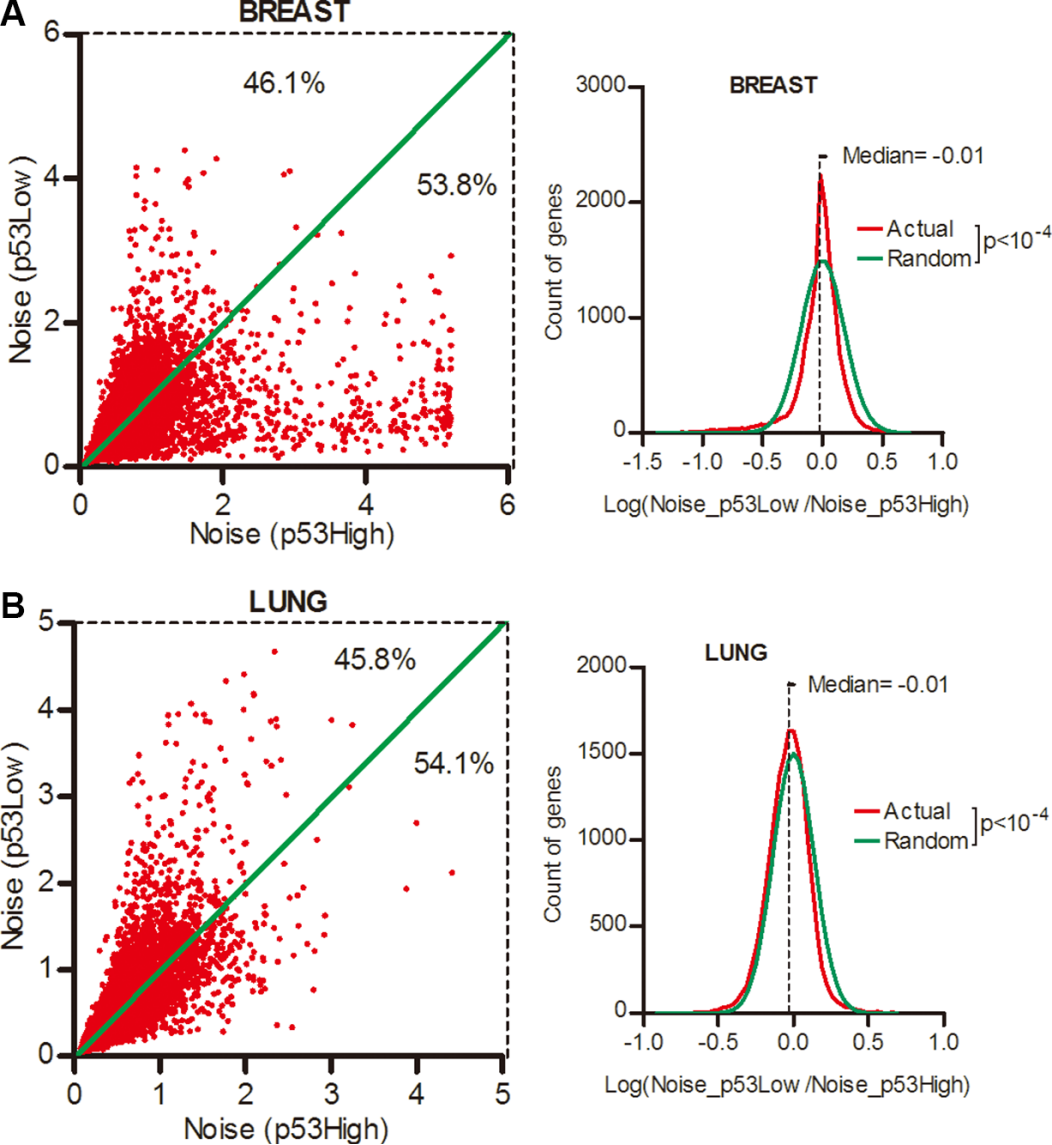
Gene expression noise was positively correlated with p53 activity in normal tissues Normal tissues from breast (**A**) and lung cancer (**B**) patients were sorted into the top and bottom quartiles of p53 activity. The expression noise was calculated in each quartile. The frequency of Log (Noise_p53Low/Noise_p53High) was plotted in contrast to random distribution. Wilcoxon's signed rank test was used to test whether the median of Log (Noise_Tumor/Noise_Normal) equals to 0.

We next asked whether p53 pathway played a role in the expression noise control in normal tissues. To answer this question, the RNA-seq data of the normal tissues of breast and lung cancer patients was used. We sorted patients into the top and bottom quartiles according to the p53 activity (Supplementary Table S7) and surprisingly found that there were only 46.1% and 45.8% genes had increased noise in breast and lung tissues of lower p53 activity groups when compared to higher p53 activity groups (Figure 7A and 7B, Wilcoxon's signed rank test *p* < 0.0001). Hence, in contrast to cancer tissues, gene expression noise was positively correlated to p53 status in normal tissues.

## DISCUSSION

Expression noise has been studied experimentally in a variety of cells, ranging from bacteria to mammalian cells with most of the attention has been restricted to intracellular noise in simple systems, such as genetic circuits, or a connected set of cellular reactions [25, 26]. At population level, gene expression noise study is relatively lack. In this work, by using the RNA-seq data we studied the change of gene expression noise in different human cancer types at whole genomic level. Genome instability is recognized as one of the hallmarks of cancer and multiple levels of gene regulations are dysfunctional due to genetic and epigenetic changes [21], which may increase the intrinsic noise of gene expression in cancers. Indeed, we found that expression noise of most genes was increased in cancers as compared to adjacent normal tissues in BRCA, LIHC, LUAD and LUSC, and to a lesser extent in COAD. Moreover, the gene expression noise was significantly increased in advanced stage cancers when compared to early stage cancers. These results showed a dynamic loss of expression control as disease progressing. Based on these findings, we suggested that cancer patient may benefit from therapies aimed to reduce gene expression noise.

Tumor suppressor p53 plays an important role in DNA damage response and genome stability surveillance. We showed that more than 60% genes had increased expression noise in patients with lower p53 activity in breast, liver, and lung cancers, implying that loss of p53 function could increase gene expression noise. The mechanism that p53 inhibit gene expression noise is not completely known in so far. While inducing cell cycle arrest, Demidenko and other researchers showed that p53 simultaneously suppressed cell senescence program [27–30]. Senescence was an integrated and widespread component of cancer development [31]. Insterestingly, increased gene expression noise was observed in aged mouse cardiomyocytes [32]. Thus, p53 may inhibit gene expression noise in cancers partly through its anti-senescence function. However, the relationship between p53 activity and expression noise in cancer was not observed in normal breast and lung tissues. One possible explanation for the difference is that DNA repair apparatus like p53 pathway remains at extremely low levels in physiological conditions [33]; and elevated p53 activity may implicate pathological changes in the peri-tumor tissues.

Tumor-infiltrated immune cells are frequently observed in cancers. Depending on the type of the immune cells, they exert supportive or suppressive roles in cancer development. Anti-tumor immune activity was mainly mediated by infiltrated cytotoxic T lymphocytes and natural killer cells through secretion of Granzyme and Perforin [34, 35]. We found that higher local immune activity (as measured by the mRNA levels of Granzyme A and Perforin-1) predicted smaller expression noise in cancers. Effectively mobilized immune activity imposes selection pressure on cancer cells, results in elimination of cancer heterogeneity and may explain the function of immune activity as an extrinsic factor in shaping gene expression noise in cancers. Unlike in breast, liver and lung cancers, gene expression noise in colon cancer are smaller in patients with lower p53 and local immune activity. The mechanism underlying this difference will need further studies. For example, other intracellular molecules, signal pathways and extracellular factors may be involved in gene expression noise regulation.

Here we investigated the gene expression noise in human cancers at transcriptomic and populational level. Translational and post-translational control of gene expression noise in cancers was not determined. Moreover, the association of intra-tumor gene expression noise with p53 status, immune activity and its relationship with prognosis was not answered. Recent progress in single cell RNA sequencing will provide invaluable tools to solve this problem [36, 37].

## MATERIALS AND METHODS

### Datasets

The RNA-seq data (level 3) of breast invasive carcinoma (BRCA), liver hepatocellular carcinoma (LIHC), lung adenocarcinoma (LUAD), lung squamous cell carcinoma (LUSC) and colon adenocarcinoma (COAD) were downloaded from TCGA website (https://tcga-data.nci.nih.gov/). For each sample, the mRNA expression levels were determined on Illumina HiSeq 2000 RNA Sequencing Version 2 platform. The patients' IDs used in this study were listed in supplementary materials.

### Calculation of gene expression noise

Gene expression noise was defined as Standard Deviation divided by Average according to Schmiedel's method [13]. For each of a total of 16,424 genes, the noise was calculated in the normal and/or tumor tissues of a patient cohort. The logarithms of Noise_Tumor/Noise_Normal were used to compare its distribution from random.

### Gene ontology analysis

Gene Ontology and Pathway analysis were performed on The PANTHER Classification System (http://pantherdb.org/). This platform provides a comprehensive set of functional annotation tools to understand biological meaning behind large lists of genes.

### Metrics of p53 status and local immune activity

As described by Cristescu et al., the p53 status in a tumor sample was represented as the geometric mean of the mRNA levels of CDKN1A (also known as p21) and MDM2, two key molecules that involved in p53 pathway [38]. Similarly, the tumor local immune activity was calculated as the geometric mean of Granzyme A and Perforin-1 mRNA levels according to Rooney's method [39]. Patients were then sorted by their p53 or immune activity, and the difference of gene expression noise between the top quartile (higher p53 or immune activity) and bottom quartile (lower p53 or immune activity) patient groups was investigated.

### Statistics

Data analysis was performed with Graphpad software. Wilcoxon's signed rank test was used to test whether the median of a set of values equals to zero (two tailed).

## SUPPLEMENTARY MATERIALS FIGURES AND TABLE


