# Supplementary material for: Increased gene expression noise in human cancers is correlated with low p53 and immune activities as well as late stage cancer
Source: Oncotarget. 2016 Oct 4;7(44):72011–20. doi: 10.18632/oncotarget.12457 (PMC5342140; doi:10.18632/oncotarget.12457)
Supplement: Supplementary file 1 [file oncotarget-07-72011-s001.pdf]

Increased gene expression noise in human cancers is correlated with low p53 and immune activities as well as late stage cancer

Supplementary Materials

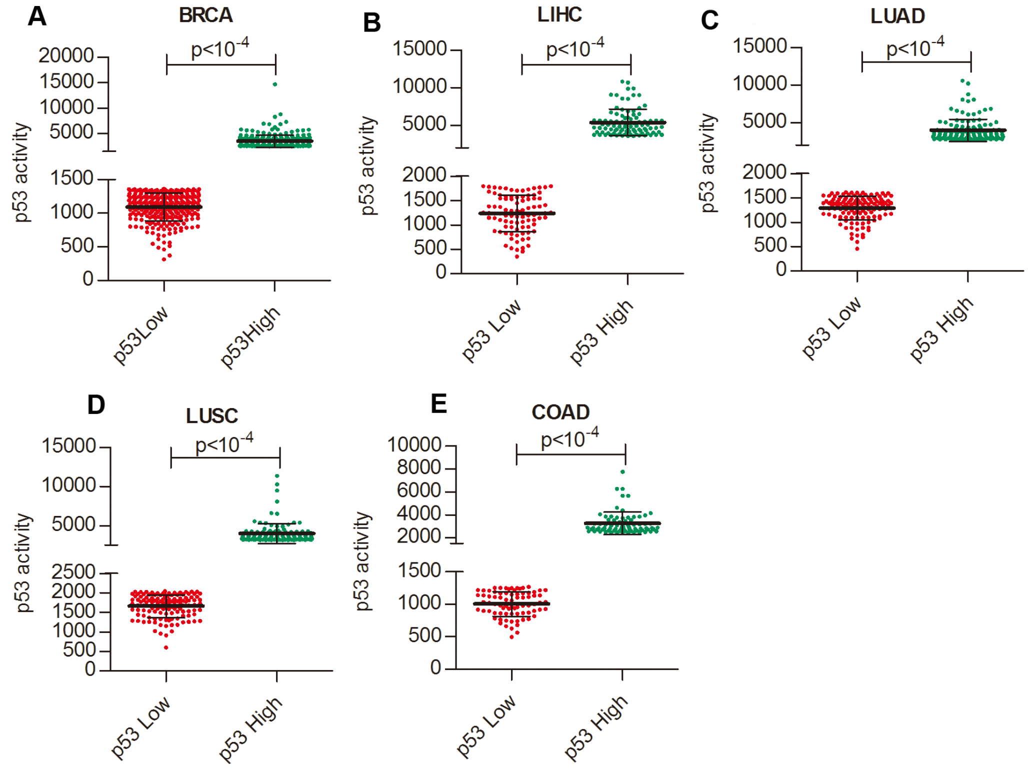

Supplementary Figure S1: p53 activity in the top and bottom quartiles of patient cohorts related to Figure 4. p53 activity was calculated as the geometric mean of the mRNA levels of CDKN1A(also known as p21) and MDM2.

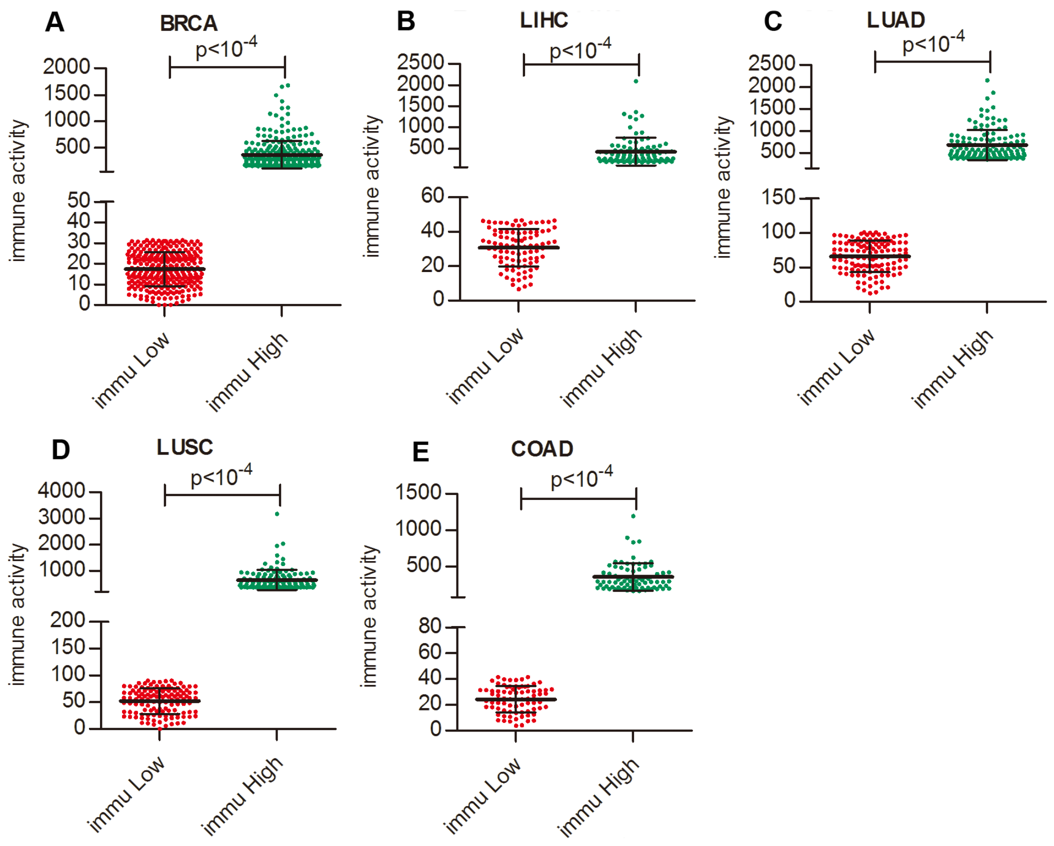

Supplementary Figure S2: Local immune activity in the top and bottom quartiles of patient cohorts related to Figure 5. Immune activity was calculated as the geometric mean of Granzyme A and Perforin-1 mRNA levels.

Supplementary Table S1: Patient ID list related to Figure 1. See Supplementary_Table_S1

Supplementary Table S2: Patient ID list related to Figure 2

BRCA patient ID list related to Figure 2A

patient ID	Tumor nuclei percent	patient ID	Tumor nuclei percent
TCGA-A7-A0CE	80	TCGA-BH-A203	90
TCGA-A7-A0CH	95	TCGA-BH-A204	90
TCGA-A7-A0D9	95	TCGA-BH-A208	80
TCGA-A7-A0DB	95	TCGA-BH-A209	80
TCGA-A7-A0DC	90	TCGA-E2-A158	85
TCGA-A7-A13E	80	TCGA-E2-A15K	90
TCGA-A7-A13F	90	TCGA-E2-A15M	90
TCGA-AC-A2FM	95	TCGA-E2-A1BC	85
TCGA-BH-A18K	80	TCGA-E2-A1IG	90
TCGA-BH-A18M	80	TCGA-E2-A1L7	80
TCGA-BH-A18P	80	TCGA-E2-A1LB	90
TCGA-BH-A18Q	80	TCGA-E2-A1LH	80
TCGA-BH-A18U	80	TCGA-E2-A1LS	80
TCGA-BH-A1EO	80	TCGA-E9-A1N4	90
TCGA-BH-A1EU	90	TCGA-E9-A1N5	94
TCGA-BH-A1EW	90	TCGA-E9-A1N6	85
TCGA-BH-A1F0	80	TCGA-E9-A1N9	93
TCGA-BH-A1F2	80	TCGA-E9-A1NA	95
TCGA-BH-A1F8	80	TCGA-E9-A1ND	90
TCGA-BH-A1FB	80	TCGA-E9-A1NF	87
TCGA-BH-A1FC	80	TCGA-E9-A1NG	90
TCGA-BH-A1FD	80	TCGA-E9-A1R7	95
TCGA-BH-A1FG	80	TCGA-E9-A1RD	92
TCGA-BH-A1FM	80	TCGA-E9-A1RI	100
TCGA-BH-A1FN	80	TCGA-GI-A2C9	90
TCGA-BH-A1FR	90		

BRCA patient ID list related to Figure 2B

patient ID	Tumor nuclei percent	patient ID	Tumor nuclei percent
TCGA-A7-A0DC	90	TCGA-E9-A1NG	90
TCGA-A7-A13F	90	TCGA-GI-A2C9	90
TCGA-BH-A1EU	90	TCGA-E9-A1RD	92
TCGA-BH-A1EW	90	TCGA-E9-A1N9	93
TCGA-BH-A1FR	90	TCGA-E9-A1N5	94
TCGA-BH-A203	90	TCGA-A7-A0CH	95
TCGA-BH-A204	90	TCGA-A7-A0D9	95
TCGA-E2-A15K	90	TCGA-A7-A0DB	95
TCGA-E2-A15M	90	TCGA-AC-A2FM	95
TCGA-E2-A1IG	90	TCGA-E9-A1NA	95
TCGA-E2-A1LB	90	TCGA-E9-A1R7	95
TCGA-E9-A1N4	90	TCGA-E9-A1RI	100
TCGA-E9-A1ND	90		

Supplementary Table S3: Gene symbol list related to Figure 3. See Supplementary_Table_S3

Supplementary Table S4: Patient ID list related to Figure 4. See Supplementary_Table_S4

Supplementary Table S5: Patient ID list related to Figure 5. See Supplementary_Table_S5

Supplementary Table S6: Patient ID list related to Figure 6. See Supplementary_Table_S6

Supplementary Table S7: Sample ID list related to Figure 7. See Supplementary_Table_S7
